# Depression in cancer patients: a critical review

**DOI:** 10.1186/1745-0179-3-2

**Published:** 2007-02-08

**Authors:** Massimo Pasquini, Massimo Biondi

**Affiliations:** 1 Department of Psychiatry and Psychological Medicine, University "La Sapienza" of Rome, Italy; 2 Dipartimento di Scienze Psichiatriche e Medicina Psicologica. Viale dell'Università 30 – Roma, 00185, Italia

## Abstract

Cancer patients experience several stressors and emotional upheavals. Fear of death, interruption of life plans, changes in body image and self-esteem, changes in social role and lifestyle are all important issues to be faced. Moreover, Depressive Disorders may impact the course of the disease and compliance. The cost and prevalence, the impairment caused, and the diagnostic and therapeutic uncertainty surrounding depressive symptoms among cancer patients make these conditions a priority for research. In this article we discuss recent data, focusing on detection of Depressive Disorders, biological correlates, treatments and unmet needs of depressed cancer patients.

## 1. Background

Cancer accounts for nearly 25% of deaths in the United States, exceeded only by heart disease [1]. Major Depressive Disorder accounts for 4.4 percent of the overall global disease burden, a contribution similar to ischemic heart disease or diarrhoeal disease [2], and the Disability Adjusted Life Years (DALYs) for depression in the near future will be greater than cancer or HIV related disease [3]. A strong body of evidence demonstrates the coexistence of depression and cancer, reported prevalence rates of depression for solid tumours ranging from 20 to 50%. These results generally include all the depressive disorders not just the Major Depressive Disorder. Grassi and Rosti found that out of 201 recently diagnosed cancer patients, 15% met the criteria for a Major Depressive Disorder [4]. Sharpe estimated at 8% the prevalence of Major Depressive Disorder in a large sample of cancer patients, although most of them had an inactive disease [5]. In a sample of haematological inpatients prevalence of Major Depression was 9% [6], the same reported by Coyne et al among breast cancer patients [7]. In the screening program conducted by Ell and colleagues among low-income women with cancer the prevalence rate for Major Depression was 24% [8]. There is reason to believe that many patients affected by subclinical depression go undetected. These estimates are made around time of cancer diagnosis. More research is needed to establish the prevalence of depressive disorders in the years after the diagnosis using standardized diagnostic criteria. The prevalence of Major Depressive Disorder at time of the first breast cancer recurrence was estimated at 22% by Okamura [9]. In a recent observational cohort study, conducted among 222 women with a diagnosis of early breast cancer, prevalence of depression and anxiety was 33% at diagnosis, 15% after one year, and 45% after diagnosis of recurrence [10].

The reported variability in prevalence is attributable to several factors: medical, personal and factors related to different study methods, instruments and procedures. Not all cancer patients are the same: cancer site, age, disease stage, time from diagnosis, cancer treatment, are among the most important variables associated with prevalence of depression among cancer patients and these should be taken into account. Psychiatric morbidity and other psychological aspects among breast cancer patients are very well studied, while fewer reports regard cancer of other sites. [6,11-13]. Personal variables such as cultural and ethnical characteristics, religious attitudes, social economic status, personality traits, coping styles, social support, distance from hospital are also very important. Not all trials have taken into account the different stages of the cancer process. The importance of these variables has been recently confirmed by results obtained from an observational study, conducted on 2.600 women treated for early breast cancer, showing that depressive symptoms were not associated with objective cancer-related factors [14]. In the absence of sufficient formal trial data in cancer patients, clinical decision should be influenced by experience in the general population, and naturalistic studies appearing in cancer literature [15].

Over the last years many efforts have been made in order to improve screening procedures, but at present no standardized approach exists for the diagnosis of depression among cancer patients. An additional source of variation is represented by the between observer variability; but, perhaps, in practice, what really matters and makes the difference is the professional background, with particular reference to knowledge in psychopathology and clinical experience. Inadequate diagnosis of Major Depression among cancer patients causes reduction in quality of life, prolonged hospitalization and higher rates of non compliance with treatment plan [16]. In addition social support has been identified as an important factor alleviating depression in cancer patients, consequently, family counselling should be usefully employed to educate the family on depression that accompanies cancer [17].

## 2. Assessment of depression among cancer patients

In the field of psycho-oncology the widely used instruments BSI (Brief Symptoms Inventory) [18] and the HADS (Hospital Anxiety and Depression Scale) [19] are employed for detecting psychological distress. Such scales were specifically developed to identify symptoms in non psychiatric medical outpatients. These instruments contributed to the recognition of psychological suffering in different settings, and improved psycho-oncology. Nevertheless too often prevalence of psychiatric disorders is reported only on the basis of studies using HADS and BSI. In many studies the CES-D (Centre for Epidemiological Studies-Depression) [20] or the BDI (Beck Depression Inventory) [21], showing acceptable sensitivities and specificities in samples of cancer patients, were employed.

On the other hand, patients with symptoms of depression may not fulfil the DSM-IV criteria for Depressive Disorders [22]. This is a crucial point. As an example, the reported high rate of Adjustment Disorders is due in part to the special status of being a cancer patient, and in part to the inadequacy of DSM-IV criteria for such patients, a sort of makeshift solution.

Moreover the prevalence of depression among cancer patients is often underestimated, because many symptoms of depression, such as fatigue, weight loss, loss of appetite or sleep disruption, closely mirror the physiologic effects of cancer; or the use of certain treatments and other symptom management medication may preclude an appropriate diagnosis. [18]. As suggested by some authors [23] it is necessary to adopt an adequate approach for discriminating the somatic symptoms of cancer and those due to treatment from the criteria of DSM-IV for major depression. Three models were proposed: the inclusive ones, which suggest that somatic symptoms are counted regardless of the cause; the substituted ones, which suggest that non somatic symptoms are substituted for somatic symptoms; the exclusive ones, which suggest that somatic symptoms may be disregarded as diagnostic criteria [17]. As an example of the exclusive approach, it has been proposed to remove fatigue from the DSM-IV criteria. In our opinion the modified DSM-IV approach to diagnose Major Depression proposed at the Memorial Sloan-Kettering Cancer Centre, that eliminates anorexia and fatigue from the list of the nine criteria and requires only four of the remaining seven symptoms for diagnosis, could increase the specificity of the diagnosis [6]. Nevertheless there are still no data confirming the validity of these models.

It is to note that, as Bailey suggested [17], culture may influence symptom expression, as an example individuals of the Asian culture suffering from depression tend to present somatic symptoms of depression neglecting the psychological symptoms.

Nevertheless the issue of Adjustment Disorder remains unresolved. This issue needs more attention, because there are still many clinicians that erroneously consider Adjustment Disorder always as a normal transition. The real problem is that cancer related stressors persist for long time, so that diagnosis should be modified and, treatments are delivered with further delay.

Recently several multiphasic screening studies to detect depression have shown an increase in sensitivity and specificity of the diagnosis [5,24,25].

Another issue in detecting depressive disorders among cancer patients is the methodology of the interview. While administering rating scales in a waiting room or interviewing patients over the telephone offer practical advantages such as simplicity, low cost, and applicability to large samples, there is no doubt that a comprehensive psychiatric assessment is warranted to diagnose a Major Depressive Disorder, and this is difficult without seeing the patient. Moreover, patients need time and space. Most important data are often obtained in the last minutes of an hour visit in a dedicated room. And this has to do with relationship, not with methodology.

## 3. Features of depression

To better understand the complex nature of depression in cancer patients, it is useful to consider several aspects. A fundamental issue is that depressive disorders are syndromes not diseases, and include a variety of mood disturbances and clinical presentations. Negative mood along with low energy, poor concentration, loss of interests, memory disturbances, low self-esteem, guilt feelings, hypochondriac preoccupation, sleep and appetite disturbances and hopelessness are the most common symptoms of Major Depressive Disorder. Features underrepresented in endogenous depression, such as psychic and somatic anxiety, somatic complaints are more common in atypical depression. However, a wide variety of clinical pictures can actually be observed in depressed patients. As an example, current psychiatric classification systems tend to underestimate the role played in depressed patients by symptoms such as anger, irritability, and hostility [26,27].

As an alternative model in the assessment of depressive disorders many authors promoted a clinimetric approach rather than the psychometric model; this because in clinical practice, psychiatrists may weigh factors such as the progression of disease, the overall severity of the disorder, the patients' social support and their adaptation and resilience and reaction to stressful life circumstances [28,29]. Physicians may find this model useful and practical in the assessment of depressive symptoms among cancer patients. Coping strategies, feelings of loss, cognitive adaptation and transition are all fundamental psychological aspects that affect different clinical manifestations of every single cancer patient [30]. These components vary tremendously whether you are assessing a female with breast cancer diagnosed and treated ten years ago, or if you are dealing with a female with colon cancer, recently stomized.

Another question has to do with fatigue, the most frequently reported side effect of cancer treatment, with a prevalence ranging from 25% to 99%[31]. However, fatigue is also a feeling state not related to cancer treatments. Even though fatigue may be associated with depression in patients treated with chemotherapy, too often is taken for depression [32,33]. Moreover, in a randomized double/blind trial Morrow demonstrates that paroxetine affects depressive symptoms but not fatigue [34]. In fact, fatigue due to chemotherapy does not include an emotional component while fatigue related to a depressive syndrome does, obviously an individual in chemotherapy experiencing fatigue can develop feelings of demoralization. Bower found that in breast cancer patients not in chemotherapy, fatigue was associated with flatter cortisol slope, what may reflect a HPA axis alterations. [35]. To develop cancer is a very traumatic experience for patients, facing the possibility of death, changes in the body image, fear of invasive treatments and pain, changes in their role in the family, in their work environment and in the society at large, all representing sources of acute stress. Stress is defined by stimuli (stressors), subjective reports of an experience, a general non-specific increase in arousal, and the feedback to the brain from this response.

Fortunately many types of cancer are becoming chronic conditions. But unfortunately humans, like other animals, are not prepared to deal with prolonged stressors. Those familiar with these patients know how their organizers are full of follow-up visits and check-ups for a long time. Talking with them you can learn how these individuals view their time. It is difficult to generalize depression in such context. In this prospective we could consider some manifestation of psychological suffering as a psychoneuroendocrinal alteration. In addition, the behavioural alterations that develop during illness are not just the result of a decreased ability to react to the external world and engage in physical activities. Pro-inflammatory cytokines produced by peripheral immune cells could function as a motivational signal that tells the brain to change the organism priorities in face of the threat represented by danger signals. This reorganization of priorities results in changes at the subjective, behavioural and physiological levels [36].

## 4. Biological correlates

There have been several studies of psychoneuroimmunological mechanisms and cancer. Biological alterations were investigated in relation to coping styles, psychological status, social support and survival. Most of these studies were conducted among patients affected by breast cancer, given that for this disease survival at five years is 88% [37].

Increasing evidence has suggested that circadian patterns of cortisol secretion are altered in cancer patients with advanced disease [38-40]. Abercombie and colleagues showed that women with metastatic breast cancer had significantly flatter diurnal cortisol rhythms as compared with healthy controls [41]. Sephton found that flattened diurnal cortisol slopes were associated with decreased survival time among women with metastatic breast cancer [42], while Osborne's study found no evidence that cortisol and prolactin levels were associated with immune or psychosocial variables in breast cancer patients [43], similar results were obtained in a largest controlled study [44].

An important finding is that cortisol level in metastatic breast cancer is related to the quality of social support, belonging, appraisal, and tangible support [45].

Only one randomized controlled study showed that levels of omega-3 fatty acid are associated with minor (but not major) depression in lung cancer patients, unfortunately psychiatric diagnosis was made using HADS [46].

There are still inconsistent data regarding the association between mood disorders, NK cells, cytokines, cancer development and survival [47-50]. In 116 breast cancer patients exposure to stress was associated with lower NK cell lysis and diminished responsiveness of NK to rIFN-γ [51]; while among men affected by prostate cancer optimism and less anger suppression was associated with greater natural killer cell cytotoxicity [52]. Consistently a structured group therapy in patients affected by malignant melanoma reduced distress and showed increases in NK cell cytotoxic activity as compared to controls [53]. Musselman first, reported that higher than normal plasma Il-6 levels were associated with a diagnosis of major depression in a small sample of cancer patients [54]. This finding was confirmed in a larger sample by Jehn et al., therefore the authors proposed the IL-6 increased plasma concentration as a biomarker of depression among cancer patients [55].

Costanzo et al. found that among 61 patients with advanced ovarian cancer social support play a protective role with respect to IL-6 elevations, and IL-6 may be an independent marker of health-related quality of life [56]. Raison and Miller reviewed the role of cytokines in cancer patients and suggested that inflammation provides a physiologic substrate that promotes mood disorders [57].

In spite of the increasing evidence that IL-6 and other pro-inflammatory cytokines may play a role in the pathophysiology of mood disorders and cause behavioural and neuroendocrine consequences, in cancer patients these phenomena are rather more complicated. In fact, different data regarding the relation between IL-6 and cancer are known. Consistent results have been shown that IL-6 is a pleiotropic regulator of prostate cancer cell growth, and serum IL-6 levels has prognostic significance in patients with metastatic prostate cancer [58,59]. Moreover IL/6 induced basic fibroblast growth factor dependent angiogenesis in basal cell carcinoma [60]. In addition IL-6 may be involved in tumor-host interactions potentially favouring uveal melanoma growth, survival and proliferation [61]. Recently Berger reported that the gene encoding the IL6 is a susceptibility factor affecting racial and ethnic differences in breast cancer survival. [62]. All these data suggest that the general assertion regarding IL-6, depression and cancer needs great caution. Besides, it is well known that proinflammatory cytokines may cause depression or sickness behaviour and that psychiatric interventions affect levels of pro-inflammatory cytokines. A randomized study on the efficacy of Cognitive-Behavioral Therapy in insomnia secondary to breast cancer provides evidence that treating insomnia can alter cytokine production [63]. In summary, apathy, social isolation, sleep disturbance, fatigue, anorexia, weight loss, cognitive disturbance, decreased libido and psychomotor retardation are symptoms of both Major Depression and cytokine-induced sickness syndrome, whereas guilt, depressed mood and suicidal ideation are more common in Major Depressive Disorder.

Neuroimaging correlates are emerging. Matsuoka demonstrated that cancer survivors with intrusive recollections had a significantly smaller total amygdala volume as compared with the total amygdala volume in cancer survivors without intrusive recollections [64]. In a case control study, Yoshikawa reported an alteration in amygdala volume associated with depressed mood after cancer diagnosis in 51 cancer survivors. [65]. Data obtained using PET in depressed cancer patients showed cerebral functional alterations similar to those founded among primary depressed patients [66,67].

## 5. Depression as a risk factor in the prognosis of cancer

Data regarding the influence of psychological factors on the prognosis of cancer are available. Psychological status seems to predict the length of survival in several types of cancer such as melanoma, non-small-cell lung cancer, breast and kidney cancer. Longer follow-up indicate that a high fighting spirit confers no survival advantage, while in patients who were disease-free at 5 years, their baseline helpless/hopeless response still exerted a significant effect on disease-free survival beyond 5 (and up to 10) years. The effect is therefore maintained for up to 10 years of follow-up [68]. These results were usually obtained by validated instruments like the General Health Questionnaires, the Quality of Life Index or the Mental Adjustment to Cancer.

However there are few data regarding the unique role of mood in predicting survival. Results from 8-year follow-up study among 10.000 patients demonstrated that the coexistence of cancer and depression is associated with an increased risk of death [69].

Prieto, in the largest oncologic study that used standardized psychiatric criteria, found that among 199 hematologic cancer patients after stem-cell transplantation, Major Depressive Disorder predicted higher 1- and 3-year case-fatality-rate, and there was a trend for patients with minor depression to survive longer than patients with no depression [6]. Faller reported that in lung cancer patients emotional distress and depressive coping style predict short survival [70]. Recent studies found that high scores on a "minimizing the illness" scale predict longer survival [71,72]. Although "minimizing the illness" coping style may have clinical utility, so far this construct has not been studied in relation to mood disorders. The impact of depression on mortality has not been definitively shown. Spiegel and Giese-Davis reviewed the link between depression and cancer progression, and suggested that there is not strong evidence supporting a link of depression with cancer incidence, though some methodological problems may have affected such results [73]. However untreated depressive disorders may be linked to faster progression.

The interpretation of these results is not univocal: depression may have a direct neuroimmune effect, or depressed patients may show poorer adherence to cancer treatment, or depression related behaviours affect several aspects of patients life such as health status, quality of life, parental role, working role. Results as those obtained by Fawzy on malignant melanoma in a 10-year follow-up study, suggest that a brief structured psychoeducational group intervention can moderately affect survival. [74].

Nevertheless, examining the effect on survival of psychotherapy, Palmer and Coyne correctly noticed that studies in which survival was not designated as a primary outcome cannot be given the same weight as studies designed with survival as an a priori end point [75].

## 6. Treatment

The single most important factor precluding treatment among depressed cancer patients is the misconception that for such patients being depressed is normal. Moreover most oncologists are unfamiliar with depressive disorders. Emerging data confute the notion that depression is inevitable and untreatable [76]. Therapeutic nihilism is not justified anymore. The evidence-based health care (screening and selective treatment) currently delivered to depressed cancer patients in selected oncology centres should be widespread. A range of options for service development should be outlined, rather than just one preferred method of service delivery. The training needs of staff must be fitted into the service model, as opposed to their training experience dictating the shape of the service, as it has happened in the past [77]. Unfortunately few data from clinical trials are available on the effectiveness of treatment for depression among cancer patients [78].

### 6.1 Pharmacological treatment

Fisch provided a brilliant review of the treatment of Depressive Disorders among cancer patients, in which he focused on the inadequate quality of research in this field [79]. At present there are only 10 randomized trials comparing antidepressants to placebo. An explanation, suggested by Fisch, is the relative aversion of patients and family members to the placebo controlled study design.

One could argue that the lack of Randomized Controlled Trials is the cause of under treatment of such disorders. There are also doubts about the physicians rigorous clinical evidence based prescriptions. Furthermore, as shown by Ashbury in lung cancer patients there is a weak correlation between detection of depressive disorders and treatment [16].

Another important issue is that of the heterogeneity of the outcomes, reflecting the same problems of assessment, and therefore data are difficult to compare. It should also be noted that there are several open or uncontrolled studies suggesting potentially improvements in depressed cancer patients. Probably, thank to the clinical experience with new antidepressants effectiveness, in the near future we could have more RCT data. In addition antidepressants have potential usefulness for the management of symptoms and conditions other than depression, such as hot flashes and pain [80-83], so they are often prescribed to cancer patients [84,85].

However, prescribing antidepressants to cancer patients requires specific knowledge, experience and caution. One must take into account: the anticholinergic side effects of tricyclic antidepressants, the pro-emetic effect of SSRIs and their potential effects on the pharmacokinetics of other drugs, and the specific syndromes occurring in combination with chemotherapy [86]. Medication should be tailored to each cancer patient based on the different characteristics of the various drugs [87-89]. We do not agree with the recommendations that oncologists should prescribe antidepressant treatments, as other authors suggested. These decisions should be the responsibility of the consulting psychiatrist, because of his/her specific knowledge. Simply adding an antidepressant to other medications differs notably from a psychiatric prescription. One implication occurs with sleep disturbances, that often mirror a depressive disorder, while patients receive unsuitable medication such as benzodiazepines. Moreover, the patient's perception of being part of an active treatment is by far different from passively waiting for the medication effect.

### 6.2 Psychological treatment

Efficacy of psychological interventions in cancer depressed patients has been demonstrated, but effectiveness study are still lacking [90]. A systematic review of psychological interventions among cancer patients identified 129 trials that involved psychosocial outcomes [91]; only 24 studies showed an advantage for the intervention in terms of the endpoint of depression [79]. Previous reviews were more enthusiastic about the benefits of psychological interventions, but their approach was less conservative, particularly regarding the outcome measures for depression [92,93]. It is difficult to compare the effectiveness of psychotherapeutic interventions when the studies include several different techniques, procedures are not clearly spelled-out, or outcomes are different. Newell and colleagues proposed ten recommendations to improve the reporting of future randomized controlled trials of psychological therapies [91]. At present most interventions are cognitive behavioural oriented. Some authors recommend a cognitive approach to the treatment of depression among cancer patients, others reported that group therapy is more effective, although these indications should take into consideration the clinical variables of every single situation. It is known that in psychotherapy research there are several methodological difficulties, and this is even more true among cancer patients. Elements of fundamental relevance of the interventions are: brief structure and a clear intervention targeting. Despite these scientific issues, the main problem is that too often providers of psychological therapies are not adequately trained in psycho-oncology, or they have an inadequate contact with the oncology staff. It is important to notice that data regarding the effect of psychological interventions on biological parameters are available in the field of psycho-oncology more than in other fields.

## 7. Unmet needs of the cancer depressed patients

In oncology settings it is emerging the notion that cancer patients should not tolerate their depressive symptoms and the importance of appropriately informing them about it. Unfortunately many issues are still unresolved. The report from the Expert Working Group of the European association for Palliative Care has stressed the major unresolved problems regarding depression and cancer [90]. Among the several recommendations provided, there are the following ones: the need for specialized mental health professionals, and their different roles depending on local circumstances; the need of a close relationship between the oncology staff and the consultation-liaison psychiatry; the concept that antidepressants should be utilized without delay once the diagnosis of Major Depression has been established. It has also been suggested that staff training in detecting Depressive Disorders may not be a sufficient condition for a better management of depressive disorders. Besides, Passik and colleagues demonstrated that rarely oncologists recognize symptoms of depression, and depressed patients referred to a psychiatrist or a psychologist represent still a little percentage of total [94]. In addition, often differences in cultural perceptions of disease are underestimated. Psychological differences by background can make a major difference in the response of individuals to disease [17]. Also compliance varies for patients in the U.S., North Europe, or other countries, such as Italy.

As reported by Ell [8], in her study among the low-income, ethnic minority, only 12% of all the women affected by Major Depression were receiving medications, while among 10% of the middle-to upper income white women who met diagnostic criteria for major depression 80% were receiving antidepressants [7]. However the Multifaceted Oncology Depression Program Intervention, organized by Dwight-Johnson and colleagues, showed that depressive disorders can be successfully treated in a public sector oncology setting serving low-income patients. This model shows the importance of collaboration between several roles at different levels: community representatives, administrative and clinical committees [76].

Another underrated aspect is that not all patients want to have their depression recognised and treated [5,79]. Various factors can explain this phenomenon, culture, patient's personality, prior experience with cancer, coping styles, gender differences. Fisch reported "that patients and their families know that a positive attitude or a "fighting spirit" is important to either the health outcome or the clinician willingness to treat the cancer aggressively, and this kind of belief can translate into a perceived risk in disclosing depressive symptoms" [79] This attitude could be related to the on-line information, more and more available without an adequate filter.

Recently it has been suggested that spirituality and spiritual coping are important to women with gynaecological cancer and that staff members should consider these issues [95]. Another important unmet need is the lack of links between psychiatric services and pain clinics; unfortunately, in many general hospitals, mental health teams have scarce resources and skill for managing patients with chronic pain. In addition it is well known that patients with depressive disorders have higher costs of general medical-care services compared with their non depressed peers. Treatment of depression may reduce these costs, in many ways including reduction of sickness-absence.

Institutions may provide new training programs as psychiatric subspecialties. Only in 2003 the American Board of Psychiatry and Neurology, trough the American Board of Medical Specialties, approved Psychosomatic Medicine as a psychiatric subspecialty. This could be due to the fact that in recent decades many diseases, including cancer, became chronic conditions, requiring more complex health care delivery.
